# Pure Altruistic Gift and the Ethics of Transplant Medicine

**DOI:** 10.1007/s11673-019-09951-z

**Published:** 2019-12-10

**Authors:** Paweł Łuków

**Affiliations:** Instytut Filozofii, Uniwersytet Warszawski, Krakowskie Przedmieście 3, 00-927 Warszawa, Poland

**Keywords:** Transplant medicine, Altruism, Anonymous donation, Gift-giving

## Abstract

The article argues that altruistic giving based on anonymity, which is expected to promote social solidarity and block trade in human body parts, is conceptually defective and practically unproductive. It needs to be replaced by a more adequate notion which responds to the human practices of giving and receiving. The argument starts with identification of the main characteristics of the anonymous altruistic donation: social separation of the organ donor (or donor family) from the recipient, their mutual replaceability, non-obligatoriness of donation, and non-obligatoriness of reciprocation on the recipient’s part. Since these characteristics are also central to typical market relations, anonymous altruistic donation not only cannot promote solidarity but may encourage proposals for (regulated) markets of transplantable organs. Thus, transplant ethics needs to be reframed. It needs to be rooted in, rather than promote, the practices of giving and receiving known to human societies. As the basis for such reframing, the idea of sharing in another’s misfortune is proposed. It relies on the human practices of giving and receiving and, with appropriate regulatory safeguards, can provide a better conceptual basis for blocking commercial exchanges of human body parts.

The concept of altruistic gift informs the ethics of transplant medicine (e.g. Uniform Anatomical Gift Act 1968; Calne 1970; Jonsen 2012). Such framing of transplant ethics has been expected to encourage organ donation, and so to increase the number of available organs without recourse to commerce in human body parts. In analogy to the arguments put forward in support of altruistic blood donation (Titmuss 1970), altruistic giving of organs has also been expected to foster social bonds and solidarity.

The altruistic gift is not, however, an ethically unproblematic foundation for transplant ethics. One of the more prominent problems is the phenomenon of the “tyranny of the gift.” Organ recipients or their family members may feel trapped by such an unrepayable debt. The value of the “gift of life” that comes with the transplanted organ cannot be matched by any form of reciprocation or gratitude and brings burdens of indebtedness and dependence (Fox and Swazey 1992, 39–42; Fox and Swazey 1974, 20–32; Siminoff and Chillag 1999). The tyranny can be further intensified by the anonymity of cadaveric donors, as required by important regulatory documents (Council of Europe 2002a, art. 23; 2002b, par. 122; World Health Organization [WHO] 2010, Principle 11), and the resultant lack of opportunity to express appreciation of the gift to the family members of the deceased donor. These difficulties are mostly seen as serious but inescapable. With the exception of supporters of commerce in human organs, the transplant ethics framed in terms of altruistic gift-giving and the accompanying mutual anonymity of donors and recipients remains unquestioned, and modest alternatives, such as sharing organs, are rare (Malmqvist and Zeiler 2016).

In what follows, I shall question the framing of transplant ethics in terms of altruistic gift. It relies on mutual anonymity of organ donors and recipients which makes their relation akin to a market encounter. Altruistic or not, anonymous gift is hardly a gift. In consequence, altruistic organ donation, as it is currently understood in transplant ethics, cannot serve as an effective conceptual barrier to commerce in transplant organs or promote social bonds and solidarity.

The argument below aims to show that altruistic giving based on anonymity, which is the core of the dominant transplant ethics, is conceptually defective, even though that ethics may encourage morally noble actions. This critical stance towards transplant ethics in terms of anonymous altruistic gift does not imply support for commerce in human body parts, or similar practices such as various forms of incentives for donation, rewarded gifts or futures in organ markets (Schwindt and Vining 1986; Cohen 1989; Hansmann 1989; Blumstein 1993; Siminoff and Leonard 1999; Delmonico et al. 2002; Giubilini 2015). It does not negate the possibility of altruistic donation or its moral value. Nor does it suggest that mutual anonymity of donors and recipients should be replaced with full disclosure. The discussion is intended to show the need for reframing of transplant ethics in a way which will make it more adequate and responsive to the human practices of giving and receiving and the widely shared moral beliefs about the immorality of trade in human organs. To give anonymity a more nuanced place in transplant ethics, the reframing starts from rethinking of the relation of organ removal and implantation to social bonds and solidarity.

The first section starts with identification of the concept of *pure altruistic gift* which shapes current transplant ethics and national and international regulations and which requires that unrelated organ donation be not for gain and the giver does not know the beneficiary or is not interested in who in particular they are. There is a number of reasons to support pure altruistic gift as the core of transplant ethics and transplant policies. Although initially they might have been introduced to reduce the problems associated with the tyranny of the gift (Fox and Swazey 1974, 32), the policies based on anonymous donation are believed to have the potential to prevent or minimize commerce in human body parts. Mutually anonymous donors (or donor family members in the case of posthumous donation) and recipients cannot initiate a commercial relation, which is typically motivated by self-interest. In consequence, the mutual anonymity of recipients and donors (or donor family) calls for altruistic motives of donors (or family members); additionally, it can be expected to foster social bonds and social solidarity.

After identifying the concept of the pure altruistic gift, its key features are explored: social separation of the donor (or donor family) from the recipient, replaceability of donor (or donor family) and recipient, non-obligatoriness of giving, and non-obligatoriness of reciprocation on the recipient’s part. On this basis, the second section investigates how the relation between the donor and the recipient, which is shaped by the idea of pure altruistic gift, makes social relations between donors and recipients “as far removed from the feelings of personal interaction as any marketplace” (Arrow 1972). To the extent to which the relation between anonymous donors and recipients is analogous to the market relation, pure altruistic gift not only does not discourage thinking about human body parts as (potential) commodities but may even encourage it.

The third section argues that relations modelled after the idea of pure altruistic gift are socially impossible if they are to be seen as belonging to the practices of giving and receiving known from anthropological literature. Pure altruistic gift is not only based on an inadequate and superficial analogy between organ donation and gift-giving (Gerrand 1994; Lauritzen et al. 2001) but also contradicts the idea of gift-giving that structures gift exchanges in human societies. Pure altruistic gift resembles a charitable donation rather than a gift in the sense and function known from anthropological literature (Douglas 1990). It also removes giving and receiving from their natural normative contexts. By contrast, practices of gift-giving in human societies occur in contexts of, and are structured by, norms which found relations between members of an ethical community who recognize them.

The fourth section is programmatic. It offers a reframing of transplant ethics which sees organ donation as a part of a larger normative context of social bonds and solidarity. It is proposed that if altruistic donation is to be possible it must rely on social bonds and solidarity, which are underpinned by socially shared moral norms and associated practices, rather than create them. On this proposal, the transplanted organ is not an object just given to or shared with others but a vector or vehicle of *sharing in* the misfortune of another human being.

## Pure Altruistic Gift

Altruistic gift-giving and the prohibition of trade in human organs are well-established in the conceptual framework which informs important transplant regulations (United States 1984; United States 1987; Council of Europe 2002a; European Union 2000, art. 3; International Summit on Transplant Tourism and Organ Trafficking 2008; WHO 2010; Nuffield Council on Bioethics 2011). They are conceptually related in many ways. One of the links is the donor and recipient anonymity which is intended to help block trade in organs and foster potential donors’ altruistic motives.

Anonymity is justified principally by the intention to limit commerce in human body parts by eliminating from transplant practices those potential donors (or their family) whose self-interest might encourage trade in human body parts. Since market transactions usually rely on interaction between sellers and buyers, mutual anonymity of the potential donor and recipient makes that interaction between them, and so commercial exchange, impossible or at least difficult. Mutual anonymity of potential donors and recipients can eliminate or reduce self-interested motives on the part of potential donors to receive payment at present or secure it to their inheritors in the future, to expect reciprocation in the future for themselves (in case they themselves need an organ) or their relatives (by securing availability of an organ to them in case they need one). In effect, potential donors (or donor family members) are more likely to be altruistically motivated.

Mutual anonymity of potential donors and recipients cannot minimize or eliminate all forms of trade in human body parts. Trafficking in human body parts, transplant tourism, and brokering on the Internet are examples of market arrangements that exist despite mutual anonymity of potential donors and recipients. Accordingly, in order to minimize or eliminate trade in human body parts, mutual anonymity of donors and recipients needs a regulatory context which can effectively block indirect commercial relations between potential donors and recipients.

The altruistic gift-giving and anonymity of potential donors and recipients, which have been described above, form a complex of ideas which can be labelled *pure altruistic gift*. It is *altruistic* giving in that it is intentional provision of products or services to another person, which is expected to benefit the recipient and is not motivated exclusively by the expectation of personal direct or indirect, material or otherwise, gain to the benefactor (cf. Delmonico et al. 2002; Ghods 2004; Epstein 2008). It is *pure* altruistic because it is not intended to benefit anyone in particular but “the anonymous other” or “unnamed stranger” (Titmuss 1970). The purity of the pure altruistic gift does not consist in the absence of self-interest on the part of the benefactor. Its purity resides in the absence of a social link between the benefactor’s motives and anyone in particular who needs the transplant. In order to be motivated to make such a gift, the benefactor must intend to promote someone else’s benefit but does not need to know the identity and circumstances of the gift recipient.

This idea of pure altruistic gift is the conceptual core of the ethics of organ transplants. The concept of pure altruistic gift (or its version) comes from Richard Titmuss’s book *The Gift Relationship*. Despite his remarks on the limits of altruism (Titmuss 1971; Titmuss 1970, 101), which will be discussed later, he believed that systems of blood collection, which are based on altruistic donation for the benefit of anonymous others, are superior to commercial systems in many ways, including advancement of citizens’ altruism by provision of the institutional environment for “the individual expression of altruism and regard for the needs of others” (Titmuss 1970, 17, 240, 254, 268). Despite the differences of the medical and ethical aspects of donation of renewable tissues and donation of non-renewable organs, his argument has been later applied both to blood collection (Keown 1997) and to organ donation (Murray 1987; Rothman and Rothman 2006; Campbell 2009; Koplin 2015). Today the view is shared by numerous transplant specialists (Shaw and Webb 2015), who see the donor as “the selfless citizen offering organs to strangers” (Joralemon 1995, 345).

There are four central characteristics of the pure altruistic gift. First, the requirement of anonymity *isolates socially* the donor and the recipient (Ohnuki-Tierney 1994, 241; Sharp 1995). It puts the identity and need of the potential recipient in the periphery of the donor–recipient relation or perhaps removes them from it altogether. Anonymity places the recipient’s need and vulnerability in the realm of generalized and unspecified patients whereas donors are put in the sphere of sources of bodily material. In this way it encourages disregard of the fact that each of them is a unique human being with their individual life, projects, and relationships. Since the anonymity of the recipient to the donor is inevitable in posthumous donation, as it cannot be known in advance who will receive organs of a particular donor, the donor anonymity brings about the anonymity of the donor family. Accordingly, pure altruistic gift requires that the recipient of an organ coming from the family’s member is abstracted into a generalized patient, too.

The organ recipient’s anonymity deprives the living unrelated donor or the dead donor family^1^ of the opportunity to recognize the recipient’s need and vulnerability situated in a particular life project. Living unrelated donors or the dead donor family rely on an abstract idea of a suffering person defined almost exclusively in medical terms. Since the specificity and uniqueness of the anonymous potential recipient is inaccessible to the potential living unrelated donor or to the deceased donor family, the medical aspects of the transplant patient do not simply mediate or accompany the social relation between the living unrelated donor and the recipient. Rather, because of the absence of more recipient-specific reasons to donate, the medical aspects of the situation of the potential recipient constitute or define the abstract relation between them.

The social isolation of the potential unrelated donor or dead donor’s family members from the recipient of a pure altruistic gift leads, secondly, to *replaceability* of the recipient and, possibly, of the donor or their family. Since the need and vulnerability of the recipient is generalized into an abstract patient who is not situated in a particular life project but defined by the medical aspects of their situation, the identity of the actual recipient of a donated organ is not relevant for the decision to donate. While the living unrelated donor’s or dead donor’s family’s perception of the recipient exclusively in terms of their medical need can be seen as commitment to equality, it can also bespeak lack of concern for the recipient *as* a unique individual. In social relations—in particular when a dire need is the issue—human beings participate as individual persons, and so as irreplaceable (the degree of irreplaceability may differ according to the kind of relation). Recognition of the potential recipient’s individuality can give them a sense of justification of receiving the life-saving organ: it is given to them in response to their unique need rather than their being like any other medically defined person. Similarly, the donor who is defined exclusively in terms of their medical suitability for donation is in principle replaceable by any other donor who satisfies the same medical criteria. The motives, beliefs, and value commitments of the living unrelated donor or of the dead donor’s family are made at best secondary, if not irrelevant, from the point of view of the recipient’s need for a part of the donor’s body. In extreme cases, the donor may be seen as just flesh which happens to be useful to the recipient.

Third, pure altruistic giving, although highly praised, is *non-obligatory*: “The act of donation should be regarded as heroic and honoured as such by representatives of the government and civil society organizations” (International Summit on Transplant Tourism and Organ Trafficking 2008; cf. Jonas 1969, 232–233; Gerrand 1994). A natural reading of such statements is that donation is a supererogatory act, or an action which is beyond the call of duty (Urmson 1958). When placed in the context of anonymity of the pure altruistic gift, donation seen as supererogatory requires that all organ recipients are to be treated uniformly and decisions to donate as equally meritorious. Accordingly, every live donation for the benefit of strangers is morally indistinguishable from donation of organs to be retrieved after the donor’s death and from live donation for recipients who are closely related to the donor. Such an interpretation conflicts with common moral beliefs. Potential posthumous donors do not need their organs after death, and so donating them for transplants—whether to strangers or close relatives—is hardly a sacrifice on their part. Close relationships usually demand more than relationships with strangers, and so they can generate obligations with regard to donation which do not obtain in other contexts.

An alternative interpretation of the non-obligatoriness of organ donation—one which will take into account the differences regarding the time of organ removal and the relations between potential donors and recipients—is to see it in the context of the duty to help those in need, that is, the duty of beneficence or charity (Gerrand 1994; Peters 1986). On this view, organ donation is not obligatory as such, irrespective of the circumstances or relationship between the potential benefactor and beneficiary, but is one of many possible ways of discharging the general duty to help. Performing such a duty on a particular occasion would remain within the agent’s discretion. Thus, the potential donor would decide who should benefit from donation and whether their organs should be removed during their life or posthumously.

This kind of duty is known to the philosophical tradition as imperfect duty (Pufendorf 1994, 106; Kant 1996 [1785], 4: 421n [473], 1996 [1797], 6: 390 [521]). Such duties leave it to the agent to decide on what occasion, and in relation to whom, the action in question is to be performed. Since the worth of the corresponding actions stems from their being voluntarily undertaken, such duties cannot be coercively enforced and performance of the corresponding actions is meritorious or praiseworthy (Hill 1971; Stratton Lake 2008). The person who fails to perform an imperfect duty is not to be blamed. The agent may have other duties to fulfil or be entitled to act for the sake of their preferences or personal attachments. For example, a parent is not only permitted but required to provide for their child first rather than for strangers; emotional bonds between life partners permit favouring taking care of a partner’s wellbeing first rather than of a co-worker’s.

In order to discharge an imperfect duty, the agent needs to know the reasons in favour of the alternative actions available on a given occasion. By contrast, anonymity of potential beneficiaries of actions makes it impossible to choose between the available courses of action. In transplants—which cover unrelated live donation and consent to retrieval of organs after the donor’s death—it implies that donation for the benefit of an anonymous stranger is at most a discretionary, and so non-obligatory, act. Donation of one’s organs does not issue from or create an ethical bond between the donor and the recipient. In effect, pure altruistic gift-giving does not respond to any particular known need of a potential beneficiary recognised as requiring action on the part of the potential donor; rather, it is a response to an imagined or possible need of an unspecified recipient, which does not command action of this particular donor. Even though posthumous donation does not involve any sacrifice on the part of the potential donor, the idea of the pure altruistic gift makes the concern for an anonymous person who is potentially in need appear as exceeding what is due to others, and as (almost) heroic. More dramatically, from the perspective of the pure altruistic gift, living unrelated donation seems incomprehensible due to the lack of an ethical or emotional link between the donor and the recipient and to the health risks to the donor (Landolt et al. 2001; Henderson et al. 2003; Drakulić and Jeger 2007).

Fourth, pure altruistic giving is accompanied by *non-obligatoriness of reciprocation*. Since the organ donor or their family are not known to the organ recipient, the latter cannot be obligated to reciprocate to them rather than to anyone else. Depending on the legal system, the recipient can be grateful to an abstract donor who (in an opt-in system) decided to donate their organs or (in an opt-out system) did not object to retrieval of their organs after death or (in a system which gives the family of the deceased a right to object to organ retrieval from their relative’s body) to the dead donor’s family members. Within the perspective shaped by the pure altruistic gift, individuals in all those categories remain anonymous and abstract to the recipient. The gratitude cannot take the form of an action towards the particular donor or their relatives. Separated by anonymity from their donor, the recipient cannot show their gratitude to the actual donor or to their family.

Since mutual anonymity of the parties of the transplant procedure makes it impossible to initiate social relationship between them, they can at best enter an imaginary, rather than real, relationship. The imaginary character of the relation is further characterized by the social and ethical discontinuity secured by lack of reciprocity. In this way the relationship between donors and recipients is fundamentally different from the practices of giving and receiving known to human societies (Mauss 1990[1925]). The recipient and the donor are thus more isolated from each other than they would be if they entered a typical market relation, where some, even if mediated (e.g., online commerce), interaction between the parties takes place.

## The Pure Altruistic Gift Relationship and the Market Relationship

The characteristics of the pure altruistic gift, which define the relation between organ donors or their family and recipients, show a striking similarity between that relationship and a market relation. An overview of the market relation will make this resemblance explicit.

To avoid misunderstandings, three points need to be made. First, the market relation to be discussed below is not identical to the relation based on the idea of pure altruistic gift. The most salient difference between them is the fact that the pure altruistic gift does not involve remuneration for, or exchange of, goods or services, which is the defining feature of market relations (Polanyi 1957). Secondly, the relationships between buyers and sellers in a market economy are not exactly the same in every society. The buyer–seller relation is shaped by the mores of the society in which it occurs. It should be no surprise to find various characteristics which are unique to the market relation of a given society. The account to be presented below describes an ideal typical seller–buyer relation of the competitive market economy which is free of monopolistic market agents. Thirdly, the ideal type market relationship to be outlined below is not generalization from empirical evidence but an element of the conceptual framework which defines markets.

As indicated, a typical buyer–seller relation in a market economy reveals resemblance of that relation to the relation between organ donor (or their family) and recipient within the perspective of the pure altruistic gift. The four characteristics of the pure altruistic gift provide an apt guidance in this respect. Markets characteristically *isolate* providers from recipients in that a typical transaction is a hands-off interaction without significant social bonds that would relate its parties (Weber 1978[1922], 635–640; Simmel 2004, 298–304; Polanyi 2001, 59–80). Marketing techniques may aim at establishing long-term relationships between businesses and their customers (cf. Customer Relationship Management) but such relations are instruments of making profit (cf. “customer value”), rather than relationships valued for their own sakes. Thus, the seller–buyer relation is characterized by *social separation* of its parties. This separation is explained by the fact that the main reason to initiate such a relation is typically the parties’ self-interest and satisfaction of their expectations. Accordingly, a partner to the market relation is seen as predominantly a means of acquisition of what is desired. Any element of the relation that goes beyond this instrumentality, including social bonds—such as those based on emotions, attachment, or sense of obligation—do not change the nature of the market relation.

A key consequence of the social isolation of the parties to a market transaction in a free and non-monopolistic economy is *replaceability* of its parties. The other party’s personal qualities—such as honesty or reliability—are important to the extent to which they facilitate or obstruct the process, or improve or damage the result, of the transaction. The identity of the other party is irrelevant from the perspective of the nature and purpose of the transaction. In the market exchanges taking place in a high-street shop (as opposed, for example, to online transactions) the parties are not anonymous to each other because their physical presence reveals to them some aspects of their identities. The parties may seek information about their potential partners in order to facilitate the transaction, but they are anonymous to each other in the sense of irrelevance of their partner’s personal qualities for the nature and purpose of the transaction. This impersonal aspect of the market relation is clearer in online shopping, where what matters is the reliability, reputation, and so on of the *website* rather than of the personal traits of the persons who own or run it. In consequence, such impersonal and anonymous partners are replaceable form the point of view of the nature and purpose of a market transaction.

Since market exchanges are based on self-interest, they are in principle *not obligatory*. The law may require individuals to sell (e.g., land needed for public facilities) or buy certain products (e.g., compulsory health insurance) but such obligatory transactions are not driven by market forces. They are warranted by socially recognized values (such as shared benefits or solidarity), which typically circumvent or counteract the market. In standard commercial relations in a non-monopolistic market economy, individuals are free to exchange goods or services. They are not required to sell their products or services or to buy what others offer. The market as such does not create an ethical bond between the potential partners, although the process of such exchanges usually is governed by ethical and legal norms.

The parties of a market transaction are *not obligated to reciprocate*. They are required to pay for the good or service received. In such transfers the goods or services to be provided and their prices are determined before the transaction, and payment is conditional on the partner’s performance. By contrast, an act of reciprocation is a fitting response to recognition of the already rendered, often unrequested, benefit (a good or service); the rendering of the benefit is prompted by recognition of a need of, or willingness to initiate a social relation with, the potential beneficiary and is not conditional on provision of something in return (even if it is expected); the benefit to be returned is not determined in advance and strict equivalence between the items exchanged is not sought. These differences between payments and acts of reciprocation show that the latter take place in a fairly complex context of norms internalized by the persons involved and social ties between them (Gouldner 1960; Kittay 1999, 67–68, 106–109; Hartley 2014). Such norms can shape market relations, but they are a facilitating factor of such transactions rather than their indispensable component.

Since a market exchange need not rely on a normative context which informs social bonds between its parties, their relation *lacks continuity* in the sense of an absence of recurrent personal interactions based on social bonds between the parties. A market relation ends at the moment the transaction has been concluded in accordance with its terms. It is a self-standing unit of social interaction in that its initiation does not require social bonds and once it has been completed there are no non-market reasons to continue the relation between the parties. There are no reasons for gratitude, with the exception of situations in which one of the parties is motivated by recognition of some need of the partner or is responding to an appeal by the other party to recognize some request. In such cases, however, the exchange is embedded in a richer social or ethical context of social bonds.

The parallels between the relation based on the pure altruistic gift and a typical market relation suggest that the pure altruistic gift is a problematic means of conceptualization of the ethics of organ transplants, promotion of social solidarity, and condemnation of markets in human body parts. In view of the chronic shortage of transplantable organs, the pure altruistic gift can even drive the thinking about transplant ethics in the direction of market exchanges (Ohnuki-Tierney 1994, 241). By placing the donor–recipient relation in the domain to which market relations belong (Scheper-Hughes 2007), the concept of pure altruistic gift can encourage proposals for (regulated) markets in transplant organs (Dworkin 1994; Epstein 1997, chap. 9–12; Radcliffe-Richards et al. 1998; Friedlaender 2002; Gill and Sade 2002; Kishore 2005; Taylor 2005; Matas 2007; Satel 2008; Boyer 2012). A regulated organ market is expected to increase the supply of organs which is the main rationale for such proposals. Such a market, it is believed, is free of the wrongs of the current organ procurement systems, which are accused of placing restrictions on the potential vendors’ autonomy by depriving them of the opportunity to make free disposals of their own organs (Dworkin 1994; Friedlaender 2002; Boyer 2012), hiding organ prices in payments for transplant services and equipment (Boyer 2012), and sustaining inequity and unfairness in wealth distributions (Kishore 2005; Boyer 2012) by providing payment to everyone except the organ donor or their remaining relatives (Boyer 2012). It is quite likely that the proposals are to a significant extent encouraged by the underlying idea of the pure altruistic gift.

## The Impossibility of the Pure Altruistic Gift-Giving

The ideal of pure altruistic gift contrasts with the anthropological data on gift-giving. Various sources show that typical gift-giving is not identical with an act of courtesy, charity, or selfless sacrifice for the sake of others; nor is it identical with market exchange. Since gift-giving did not vanish with pre-market economy, where it was the main mechanism of circulation of goods and services, it can be hypothesized that it plays and important role in the structuring and functioning of human societies independently of their economic system. Classical anthropological sources demonstrate that the ancient customs and rituals of gift-giving, gift-receiving, and reciprocation found and preserve social bonds (Mauss 1990 [1925]; Malinowski 2013 [1926]; Thurnwald 1932; Gouldner 1960; Lévi-Strauss 1969 [1947]). As before, a discussion of the nature and functions of gift-giving practiced in human societies will benefit from the guidance by the four characteristics of the pure altruistic gift relation.

One prominent characteristic of gift exchanges is that the gift-giver expects something in return from the recipient, and so such encounters are instruments of *social connectedness*. The items expected need not be of material or financial kind or value, and the reward may be the taking part in exchange (Lévi-Strauss 1969 [1947], 55–60). The agent’s motive links them with the act of giving (Siminoff and Chillag 1999) and a particular participant in the encounter. Some motives (e.g., profit making in certain contexts) or their characteristics (e.g., ruthlessness in the pursuit of one’s own interest) are unacceptable in gift-giving, but others can be legitimate. It is not self-orientedness of the agent as such that makes certain acts of gift-giving questionable, but violation of socially recognized norms.

The element of self-orientedness in gift-giving, which relies on expected reciprocation, requires that the recipient be recognizable as a unique individual with their life, projects, and relationships. If the giver is to know the what, when, and how that is expected in reciprocation, they must know and appreciate not only the recipient’s vulnerabilities, needs, and desires but also their potential to reciprocate. Thus, the parties of gift-giving are relatively *irreplaceable* to each other. The giver’s expectation cannot be met by just anyone but by someone who has particular characteristics that make them the appropriate partner of gift-giving. The factors that can make others potential recipients of gifts are both the need for what the potential giver can offer and the goods or services the recipient has at their disposal.

The normative status of gift-giving resembles that of imperfect duties. Individuals are *obligated* to give gifts, but it is up to them to decide to whom and on what occasions they will give. While one can decide not to give a gift to a particular person, it would be unacceptable to refuse to give gifts to everyone who one recognizes as in need and potentially benefitting from the gift. Publicly visible failure to give gifts would evidence unwillingness to participate in social interaction and often would practically deprive such a person of the membership in their group in the long run. Naturally, the strength of the imperfect duty to give gifts depends on various factors, among which previous gift encounters and emotional links between potential givers and recipients play central roles. Potential givers and recipients who have a history of gift encounters and those who are emotionally related have a relatively stronger claim or duty to participate in gift-giving.

Since gift-giving takes place within a network of social and moral relations, recipients are normally *obligated to reciprocate*. Although they may never be able to do so—due to the lack of opportunity or the unsurpassable value of the gift—their sense of obligation to reciprocate is proof of their recognition of the importance of the act of giving and of the gift itself. Being given a gift is to be invited to participate in a social relationship by continuing the exchange, and so preserve the relation (Kopytoff 1986). Social participation requires active engagement with others and responding to their actions, and in this way it includes reciprocation. Gift recipients engage in interactions which naturally complete their encounters with others and potentially open prospects for future encounters. A gift recipient who does not recognize their obligation to reciprocate is typically criticizable for the lack of appreciation of the gift and absence of the sense of belonging to the community.

The typical human practices of giving and receiving are reflected in the desire of many transplant patients to contact the donor family in some form (Dicks et al. 2018). Although some organ recipients prefer anonymity, others want contact with the donor family (Sharp 1995; Politoski, Coolican, and Casey 1996; Dobbels et al. 2009; Azuri and Tabak 2012). Some recipients and donor family members believe that an anonymous thank-you letter to the donor family is a sufficient form of contact (Maloney 1998; Kaba et al. 2005), whereas others want (also) a meeting in person (Albert 1998; Maloney 1998; Ono et al. 2008; Annema et al. 2014). Organ recipients who prefer contact with donor family want to show respect for the donor or their family (Annema et al. 2014) or gratitude (Fox and Swazey 1992, 41; Lewino et al. 1996; Albert 1998; Ono et al. 2008). Deceased-donor family members may desire to contact the recipient in order to witness the benefits of the transplant (Lewino et al. 1996; Albert 1998; Ono et al. 2008), have some form of relation to the deceased relative through the recipient (Fox and Swazey 1992, 44), or give meaning to the death of their loved one and complete the grieving process (Albert 1998).

To some extent, these attitudes are recognized by the policies of the transplant centres in which recipients can write anonymous letters to their living donors or the donor family (United Network for Organ Sharing [UNOS] 2018; NHS 2017) or initiate a direct contact after initial anonymous correspondence (LifeCenter Northwest 2018). Substitute forms of interaction with donors are also becoming more frequent and elaborate (Sharp 2007, 23–30). Such interactions are not without psychological and ethical risks, and so attempts to get in touch with the donor or the donor family must be handled with caution (Lewino et al. 1996; Politoski, Coolican, and Casey 1996; Albert 1998; Clayville 1999; Shaw 2012; Azuri, Tabak, and Kreitler 2013). It is advisable therefore that, if allowed, the contact be, among others, extended in time or gradual, facilitated by the organ procurement agency, and well-informed with respect to the psychological and ethical risks and potential benefits of such interactions to the parties involved (Lewino et al. 1996).

Compared with the classical practices of gift-giving and the empirical data regarding donor families’ and recipients’ desire to contact, the idea of pure altruistic gift is a social and moral impossibility. Richard Titmuss, the champion of anonymous altruistic blood donation (which relies on the idea of pure altruistic gift) whose ideas serve as support for pure altruistic organ donation, seems to have appreciated the contrasts between the actual practices of gift-giving and his proposal as well as the difficulties of the latter. In his classic book, Titmuss voiced misgivings about altruistic blood donation: “There must be some sense of obligation, approval and interest; some awareness of need and the purposes of the blood gift; perhaps some organised group rivalry in generosity; some knowledge that fellow-members of the community who are young or old or sick cannot donate, and some expectation and assurance that a return gift may be needed at some future time” (Titmuss 1970, 101; cf. 268–269). Even if reluctantly, Titmuss did recognize that actual gift-giving practices rely on actual social bonds, are embedded in a context of ethical beliefs, and generate various obligations. But, as the phrase “some sense of …” in the passage above suggests, he downplayed the need for a more robust social and ethical context provided by particular realities and individuals for gift-giving to be practiced. He believed that, rather than rely on already existing social and ethical practices and contexts of giving and receiving, the pure altruistic gift should create its own practice and context of giving and receiving.

The actual practices of giving and receiving are embedded in rich social frameworks which contain concepts and norms that define and structure behaviour. These frameworks are made, inter alia, of various ethical and behavioural norms that identify reasons for giving and define acts of giving and receiving. Such frameworks are not products of unstructured acts of giving and receiving. They *are* the context, which makes acts of giving and receiving possible, and in this way they confer social and ethical meaning to giving and receiving as expressions of moral values and commitments recognized in a society. Gift-giving and receiving can reflexively reshape the values and commitments which define them and the practices to which they belong. However, these practices do not shape the values and commitments of those who engage in them in a social or normative vacuum. Gift-giving and receiving is made possible by, and grows out of, socially recognized values and commitments. When giving of gifts is altruistic it is not purely altruistic; when giving is pure altruistic it is not giving of gifts.

## The Context of Sharing in Another’s Misfortune

It would be a gross error to model transplant ethics uncritically after the classical gift-giving practices. Some aspects of transplants do not have their counterparts in the traditional practices of giving and receiving; an alternative transplant ethics would also have to respond to potential ethical and social risks involved in transplants. The preceding discussion allows, however, for some programmatic claims about reframing of transplant ethics in a way that is informed by the human practices of giving and receiving.

The point of departure of such a reframed transplant ethics would *not* be a normative view of bodily transfers and their desirable social consequences for individuals and society but an analysis of the practices of giving and receiving understood as structured responses to human finitude, dependency, and vulnerability, which are also daily companions of human beings and become particularly salient in times of crisis, such as organ failure. An analysis of such practices would reveal their normative, psychological, and social presuppositions of giving and receiving, and—in combination with the appropriate evidence provided by medical and social sciences—would clarify the nature of the benefits, burdens, and risks of transplants. The point of arrival of such an analysis would be a reframed transplant ethics.

An adequate transplant ethics must see organ donation as embedded in, and structured by, an already existing network of moral norms and commitments, which respond to human finitude, dependency, and vulnerability. A key element of such a network is mutual assistance at times of misfortune. In this network, organ transplants are to be seen not as a self-standing practice but as a technologically contextualised assistance brought about by sharing in the misfortune of another human being (Łuków 2018). Sharing in the misfortune of a potential recipient stems from, among others, recognition of the potential beneficiary’s need and its gravity or urgency, the potential donor’s or their family member’s commitment to assist those in need, their expectation of appreciation of the assistance, and the need for continuation of the relationship between the donor or donor family and the recipient.

Seen as an element of a network of sharing in others’ lives, consent to removal of an organ (during one’s life or after death) is an act of active concern for, and participation in, someone else’s misfortune. It is an act of relating oneself now to others, the body part to be transferred being a vehicle or vector of assistive relation rather than its key “participant.” Ethically and socially, the focus of such engagement is not just the body part to be transferred or the transfer itself—although, technically, the medical procedures of organ removal and implantation are central—but primarily the other person in dire need. The transferred organ is a vehicle or vector of assistance in a way akin to that in which tools and materials were vehicles or vectors of collective work in barn raising in eighteenth- and nineteenth-century rural North America. Consistent with the classical practices of giving and receiving, the constitutive element of providing assistance is a relation to others rather than merely bringing and getting what is needed. It is about *doing* things *for* and *with* others rather than merely giving and transferring objects to them.

Such an alternative approach could have important ethical and policy consequences. They cannot be discussed comprehensively here. Two points, however, deserve attention to illustrate how the view of organ donation as sharing in another person’s misfortune would change the ethical and regulatory aspects of transplants.

First, offering one’s own body parts for the treatment of others would be a discharge of an imperfect moral duty towards other members of the community. How strong this duty would be in a particular case should depend on various factors. Since retrieval of an organ from a cadaver does not imply burdens to its source, the obligation to offer one’s own organs to be removed for the treatment of others after one’s death would be much stronger than the obligation to assist others by offering one’s body part (such as kidney or a liver section) to be removed for transplant during one’s life. Similarly, the moral obligation to assist one’s near and dear would be relatively stronger than the duty to help strangers. The reframed transplant ethics could also contain an obligation to reciprocate in a way which, not being payment, leaves room for various forms of appreciation of the benefit received. Such duties would then reflexively create and sustain wider networks of sharing in each other’s lives.

At the policy level, informed consent of the prospective living benefactor for removal of a transplantable organ would be non-negotiable if it were to be an element of assistance rather than exploitation or extortion. In the case of posthumous retrieval, consent to donation could take various forms, depending on the view of human embodiment on which public policies were based and various other factors such as the dominant cultural beliefs regarding the appropriate dealing with human remains or state interests in the body, such as those related to criminal proceedings (Łuków 2018).

Secondly, in view of the differences of opinion, transplant policies should give the benefactors and beneficiaries the opportunity to choose if they wish to remain anonymous, what information or its types should be disclosed to the other party, and what forms contacts between organ recipients and donors or donor family could and should take. In posthumous retrieval the potential benefactor could decide during their life (preferably with the assistance of the family) what information should be disclosed to the beneficiary and to what extent the family should be involved in a relation with the beneficiary after the potential donor’s death (e.g., Lauritzen et al. 2001). To minimize the risk of illicit transactions between the beneficiary and the benefactor or their family, should they decide to know each other, the beneficiary could remain anonymous to the benefactor and their family until the surgery is completed. In such cases provision of the transplant material would not be conditional on benefits from the beneficiary. Since living benefactors are predominantly patient’s family members or partners, organ removal from such benefactors would not be associated with significantly higher risk of commercial transactions.

## Conclusion

The idea of pure altruistic gift, on which important elements of transplant ethics are currently based, is anthropologically inadequate, seems socially unfeasible, and does not have conceptual resources that can block conceptually trade in human body parts. An attractive alternative to it could be an ethical framework of sharing in the misfortune of another. In such a framework, organ transplants would not be an instrument of solidarity, social bonds, and cohesion but their result. By building on the existing human potential for, and normative components of, helping those in need, a reframed transplant ethics could help build communities of mutually caring friends rather than societies of benevolent strangers.

## Footnotes

1 For the sake of brevity, throughout this paper the deceased donor’s family members are treated as actually making decisions whether organs should be retrieved from their dead relatives, despite differences between legal systems. For example, some legal systems, dead donor’s family members have the right to consent to donation or refuse it. In others, dead donor’s family do not have such a right but they are asked whether they are aware of the deceased’s objection to donation.
